# Whole-blood DNA Methylation Markers for Risk Stratification in Colorectal Cancer Screening: A Systematic Review

**DOI:** 10.3390/cancers11070912

**Published:** 2019-06-28

**Authors:** Janhavi R. Raut, Zhong Guan, Petra Schrotz-King, Hermann Brenner

**Affiliations:** 1 Division of Preventive Oncology, German Cancer Research Center (DKFZ) and National Center for Tumor Diseases (NCT), 69120 Heidelberg, Germany; 2 Medical Faculty Heidelberg, Heidelberg University, 69120 Heidelberg, Germany; 3 Division of Clinical Epidemiology and Aging Research, German Cancer Research Center (DKFZ), 69120 Heidelberg, Germany; 4 German Cancer Consortium (DKTK), 69120 Heidelberg, Germany

**Keywords:** DNA methylation, whole blood, colorectal neoplasms, odds ratio, risk stratification

## Abstract

DNA methylation profiles within whole-blood samples have been reported to be associated with colorectal cancer (CRC) occurrence and might enable risk stratification for CRC. We systematically reviewed and summarized studies addressing the association of whole-blood DNA methylation markers and risk of developing CRC or its precursors. We searched PubMed and ISI Web of Knowledge to identify relevant studies published until 12th November 2018. Two reviewers independently extracted data on study population characteristics, candidate genes, methylation measurement methods, methylation levels of patients in comparison to healthy controls, *p*-values, and odds ratios of the markers. Overall, 19 studies reporting 102 methylation markers for risk assessment of colorectal neoplasms met our inclusion criteria. The studies mostly used Methylation Specific Polymerase Chain Reaction (MS-PCR) for assessing the methylation status of a defined set of genes. Only two studies applied array-based genome-wide assays to assess the methylation levels. Five studies incorporated panels consisting of 2–10 individual methylation markers to assess their potential for stratifying the risk of developing colorectal neoplasms. However, none of these associations was confirmed in an independent cohort. In conclusion, whole-blood DNA methylation markers may be useful as biomarkers for risk stratification in CRC screening, but reproducible risk prediction algorithms are yet to be established by large scale epigenome-wide studies with thorough validation of results in prospective study cohorts including large screening populations. The possibilities of enhancing predictive power by combining methylation data with polygenetic risk scores and environmental risk factors need to be explored.

## 1. Introduction

Worldwide, colorectal cancer (CRC) is the third most common cancer in terms of incidence and second in terms of mortality, accounting for nearly 1.8 million new cases and 881,000 deaths annually [1]. The disease burden has been shown to be dramatically decreased with population-based screening, which allows early detection and removal of precancerous lesions [2,3,4]. Despite strong recommendations for screening, the participation rate in the general population remains low [5,6]. Among the several factors accounting for poor screening uptake is a lack of perceived risk among the patients and physicians [7,8,9]. Screening adherence can potentially be increased through identification of risk subgroups in the population. Risk stratification could assist both physicians and patients, in making more informed screening decisions [10]. 

Besides genetic alterations, epigenetic alterations that alter gene expression without changing the DNA sequence have been increasingly implicated in the etiology of CRC [11]. DNA methylation is an epigenetic modification of special interest as it has been identified to play a significant role in CRC initiation [12,13,14] and is therefore a potential biomarker for evaluating the likelihood of developing CRC. Studies have identified a plethora of biomarkers for early diagnosis or risk assessment of CRC using DNA derived from tissue, blood, stool, urine as well as bowel lavage fluid [15,16,17,18,19]. Among the various samples used to extract DNA, blood sampling offers the advantage of being commonly available in epidemiological studies, minimally invasive and more acceptable to the screening population [20,21]. Methylation of septin 9 (SEPT9) in tumor-derived cell-free DNA (cfDNA) is the only blood-based FDA-approved methylation biomarker for detecting CRC [22,23]. Although methylation changes can be detected in cfDNA from plasma or serum, the low proportion of tumoral DNA in cfDNA and wide variation in methods for processing blood and isolating cfDNA present significant problems [24,25,26]. Compared to DNA extracted from plasma or serum, the recovery of DNA from whole blood is more efficient. While emerging evidence also suggests that peripheral blood cell or white blood cell (WBC) DNA methylation is linked to cancer susceptibility [19,25,27,28,29,30], a comprehensive review of the potential use of DNA methylation markers in whole blood for CRC risk stratification or diagnosis has not been done yet. We provide here a systematic review of the published literature including articles that have assessed DNA methylation in whole-blood-borne DNA from patients with colorectal neoplasms and healthy controls. We report the performance of DNA methylation markers in whole blood for CRC risk evaluation

## 2. Results

### 2.1. Study Selection

**Figure 1:**
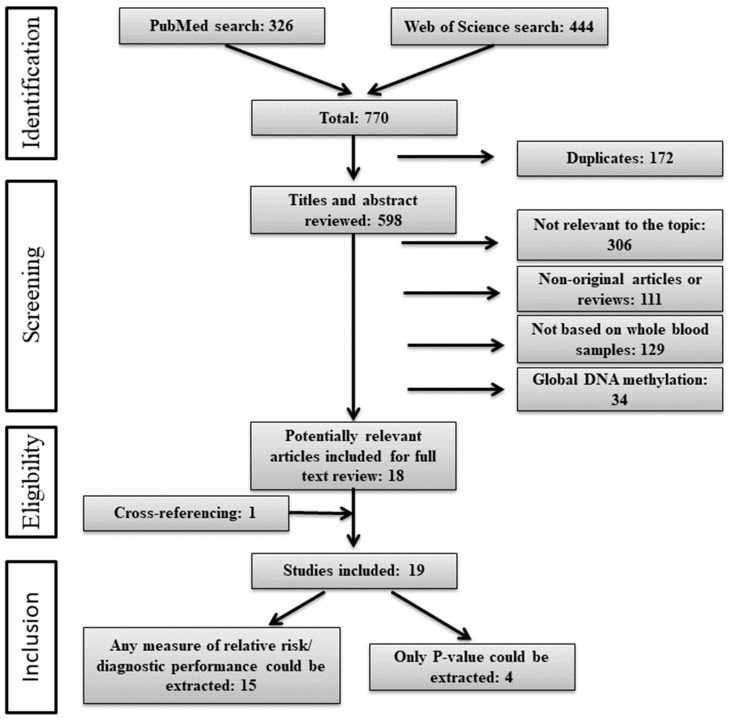
Flow diagram of the literature search process (search until 12.11.2018).

The literature search and selection process is shown in Figure 1. We identified 770 articles of which 172 were duplicates and removed. Titles and abstracts of the remaining 598 articles were reviewed, leading to 18 articles that were selected for a full-text review. Cross-referencing resulted in identification of one additional study. Finally, 19 studies met our inclusion criteria and were included in this review. Information regarding odds ratios (ORs) could be extracted in 15 articles. In the remaining four studies, only the results of the statistical tests for the differences in DNA methylation levels between cases and controls were reported. Areas under the receiver operating characteristic curves (AUCs) could be extracted from two studies only.

### 2.2. Study Characteristics

An overview on the study characteristics is shown in Supplementary Table S1. Of the 19 included studies, ten were carried out in Europe, including three studies from Italy [30,31,32], two from the United Kingdom [33,34], and one each from Germany [19], Finland [27], Sweden [28], France [35], and Lithuania [36]. Five studies were carried out in China [37,38,39,40,41], three in the United States [42,43,44], and one in Canada [45]. A majority of studies (16) followed a case-control design and collected blood samples from cases at the time of diagnosis or shortly after diagnosis. Of the 19 included studies, two studies [27,28] and one cohort from a third study [34] were nested case-control studies which allowed assessment of methylation status in cases prior to disease onset. In these studies, CRC cases were identified during follow-up of the cohorts and were compared to cohort participants remaining free of CRC. Most of the included studies included only CRC patients as cases, while three studies included only colorectal adenomas [43,44,45] and two studies [33,38] included both colorectal adenomas and CRC as separate case groups. Five case-control studies [36,37,38,41,42] explicitly reported that blood samples were collected from cases before treatment (chemotherapy or adjuvant radiotherapy), while the remaining studies did not specify treatment status at the time of sampling. In one study [19], 33 of the 93 cases were reported to have received neoadjuvant therapy before blood sampling. The age range (in one study) or average age (in 13 studies) was reported in 14 studies. Most of these studies reported a fairly similar age distribution between cases and controls. However, the mean age varied largely between cases and controls in some studies e.g., 69 versus 49 years in a study from China [38] and 64 versus 40 years in a study from France [35]. To measure methylation, most of the studies used Methylation Specific Polymerase Chain Reaction (MS-PCR). Only two studies [19,27] applied array-based genome-wide assays, such as the Illumina Infinium HumanMethylation 450K Beadchip and Illumina GoldenGate Methylation Cancer Panel I to assess the methylation levels. A majority of the studies had low case numbers and rarely implemented internal validations. None of the studies verified the reported associations in an independent study population.

### 2.3. Overview of Whole-Blood based DNA Methylation Markers

From the included studies, methylation markers with any measure of relative risk or diagnostic performance or *p*-value for the comparison of methylation levels or with enough data to calculate them were selected. Of the included 19 studies, 16 studies applied a gene-specific approach and reported 63 methylation markers, two studies [19,27] used array-based genome-wide assays and reported 28 differentially methylated gene regions, and one study [36] used pyrosequencing to analyze methylation at 11 individual long interspersed element (LINE)-1 loci and identified one differentially methylated locus. Genes/LINE-1 regions within which these markers were identified are summarized in Table 1. Nine of the 80 genes/LINE-1 regions were reported ≥2 times and the remaining genes/LINE-1 regions were only reported once. IGF2 methylation was assessed most frequently (five times), followed by APC (three times). The frequency of statistically significant findings for each individual marker ranged from 0% to 100%. Table 1 also shows the direction of methylation changes (hypo- or hypermethylation) for colorectal adenoma or CRC cases compared to controls. Among the genes which had been evaluated ≥2 times, the majority showed no difference in methylation levels between cases and controls, and only IGF2 was hypo- or hypermethylated [35,39], SEPT9 was hypomethylated [32] and WIF1 [39] was hypermethylated in cases compared to controls. Among the 71 genes/LINE-1 regions reported only once, 52 showed no association with colorectal adenoma/CRC, whereas significant hypermethylation and hypomethylation were reported among colorectal adenoma/CRC cases for 15 and 4 genes/LINE-1 regions respectively. Supplementary Table S1 presents the targeted genetic region of all reported genes/LINE-1 regions. The vast majority of the studies assessed the methylation levels in the promoter region of targeted genes, 3 studies assessed methylation levels of the differentially methylated regions (DMRs) [28,34,35] and only a few studies assessed methylation levels of the gene body [19], transcriptional start site, gene-coding area [45], and individual long interspersed element (LINE)-1 loci [36].

### 2.4. Associations of Methylation Markers with Colorectal Neoplasms

In assessing the associations between DNA methylation markers and colorectal neoplasms, four studies reported significant ORs for dichotomized methylation levels of 15 target loci (Table 2) and four studies presented significant associations for other comparisons of quantitative methylation levels of other 31 target loci (Table 3). Of the 31 target loci identified as significantly associated using quantitative methylation levels, correction for multiple testing was applied for 29 loci [19,27,36] and only four remained significantly associated after the correction. Studies which only provided *p*-values (for difference in methylation levels between cases and controls) or non-significant ORs (for colorectal adenoma or CRC risk) are shown in Supplementary Tables S2 and S3. 

The four studies estimating dichotomized methylation of 15 target loci and reporting significant associations to risk of colorectal adenoma/CRC performed methylation analysis using MS-PCR or Methylation-sensitive High-resolution Melting (MS-HRM) (Table 2) [37,39,40,43]. Most of the ORs were adjusted for vitamin C and D use or for age and BMI. In one study, hypomethylation (no methylation detected) of markers presented positive associations with CRC risk with ORs ranging from 2.9 to 11.1, while in the other three studies, hypermethylation of markers presented positive associations with CRC risk with ORs ranging from 1.72 to 16.96.

In the four studies reporting significant associations for quantitative methylation levels of target loci, methylation levels were derived by four types of methylation techniques, including Human 450K methylation array, Sequenom EpiTYPER, Illumina GoldenGate Methylation Cancer Panel I, and pyrosequencing (Table 3) [19,27,36,45]. Two studies estimated AUCs [19,36] and two studies estimated ORs [27,45]. Among the two studies estimating ORs, one study estimated ORs for tertiles of methylation levels [27], while the other study calculated ORs for quartiles of methylation levels or for an increase in DNA methylation per one standard deviation [45]. In the study estimating ORs for tertiles of methylation levels [27], the reported associations are before multiple testing correction and none of these remained statistically significant after the correction. The two studies estimating AUCs [19,36] built diagnostic models with significant markers and reported point estimates of the AUC values. In one study, the reported AUC was internally validated by bootstrapping, yielding an optimism-corrected value [19]. Two CpG sites in the promoter region of KIAA1549L were identified as markers with best performances—cg14472551 with a c-statistic of 0.72 demonstrated the best performance in screening setting, while cg04036920 with a c-statistic of 0.70 demonstrated the best performance in clinical setting. The AUC in the other study [36] was derived from the training (derivation) populations, and is therefore prone to over-optimism due to overfitting.

### 2.5. DNA Methylation Panels

Combinations of different markers were evaluated in five studies (Table 4) [19,37,39,40,43]. Two studies [19,39] reported AUC values validated internally by bootstrapping. Liu et al. [39] estimated the association for dichotomized methylation of 10 individual markers and also calculated a weighted methylation risk score (MRS) based on a panel of these 10 markers (MRS_10). Only 5 out of the 10 individual markers were significantly associated with CRC risk with ORs ranging from 2.44 (1.53–3.87) to 4.27 (1.52–12.05) (Table 2). However, MRS_10 was significantly associated with CRC risk with OR of 3.85 (2.72–5.45) and 6.51 (3.77–11.27), respectively, for MRS-Medium group and MRS-High group compared to MRS- Low group. Furthermore, the AUC for the MRS_10 model was 0.69 (0.66–0.73, *p* < 0.0001), which represented significantly higher discrimination accuracy than any individual marker. In the other study, Heiss and Brenner [19] estimated an optimism-corrected c-statistic for three individual markers (two in the promoter region of KIAA1549L and one in the body region of BCL2), and for panels built on all three markers. The c-statistic for single markers ranged from 0.57–0.72 in the screening setting and 0.64–0.70 in the clinical setting. Compared to the performance of individual markers, the marker panel barely improved discrimination with a c-statistic 0.69 in the screening setting and 0.73 in the clinical setting.

### 2.6. Quality Assessment of Studies

The results for the quality assessment of studies using the QUADAS tool are presented in Supplementary Figures S1 and S2. Patient selection was at high risk of bias for most of the studies since they used a case–control design and did not collect samples from asymptomatic cases in a screening setting. Bias assessment for the index test was unclear for most of the studies. The reasons were lack of information on whether the test was performed blinded to the results of the reference standard and if or not a pre-specified threshold was used. For most of the studies, there were no concerns of bias for the reference standard. Regarding flow and timing, 11 studies were judged as having unclear risk of bias and seven as having low risk. Applicability concerns regarding use in a screening setting were high for patient selection as most of the studies collected blood samples from symptomatic cases at the time of diagnosis. 

## 3. Discussion

In this review, we present an overview of studies exploring the association between gene/locus-specific DNA methylation status and the risk of colorectal neoplasms. We focused on studies investigating methylation markers in whole blood and identified 19 studies evaluating 102 DNA methylation markers for colorectal adenoma/CRC risk stratification. For 22% of the 102 markers significant associations with colorectal adenoma/CRC risk were reported in either univariate or multivariate regression analysis. However, the suggested associations should be interpreted with caution considering that they were mostly reported from single studies only without further validation or replication. Furthermore, in reporting the association of 102 markers with the risk of colorectal neoplasms, correction for multiple testing was applied only for 30 markers out of which only four [19,36] remained statistically significant after the correction. 

An important factor that could have affected the outcome of the reviewed studies is the study design and population. A majority of the studies followed a case-control design and recruited study participants in clinical settings. Relying on samples obtained just before or at the time of clinical diagnosis, these studies may only provide preliminary evidence for potential risk stratification or early detection. Markers identified may only be associated with symptomatic disease and therefore of limited use for risk evaluation of asymptomatic cases. When selecting cases, only 5 studies included adenomas [33,38,43,44,45] which are the precursors of most CRCs and would be most relevant to stratify risk for developing CRC. As regards controls, the inclusion criteria were different among studies. While most of the studies included only healthy controls, three studies [19,37,43] also included diseased controls i.e., carriers of hyperplastic polyps or non-digestive system disease patients, making comparison across studies difficult. Most of the studies did not clearly report if the control cohort had undergone colonoscopy. The estimated prevalence of adenomas among older adults is 16–30% [46,47]. In studies in which control participants had not undergone colonoscopy, associations could have been underestimated by including adenoma cases as controls. Furthermore, the studies involved different ethnic groups from different geographical regions, among which DNA methylation patterns are known to be highly divergent [48,49]. Markers identified in specific populations may have limited generalizability and transferability to other populations. Given such heterogeneity in study designs and cohort compositions across reviewed studies, a number of suggestions could be made for future research to obtain more realistic estimates of associations. Markers identified in clinical settings should be evaluated in large-scale prospective studies conducted in true screening settings with external validation in ethnically and geographically diverse populations.

Other potential sources of bias that affected methylation status in the reviewed studies are the variations in blood processing methods and cell population used as a source of DNA in each study. The studies collected and stored different amounts (5–20 mL) of blood samples using a variety of methods; four studies [38,43,44,45] specified collecting samples in vacutainers containing EDTA anticoagulant and three studies [28,33,40] specified storing the samples at −70 °C to −80 °C until DNA extraction. The studies then used various kits for extracting DNA from samples including QIAamp DNA Blood Mini Kit [37,39,40], PUREGENE genomic DNA purification kit [33,42,43], Wizard genomic DNA purification kit [30,31], etc. Three studies [33,37,40] specified determining DNA concentration using UV spectrophotometry, while one study [45] determined concentration using Quant-iT^TM^ PicoGreen. Of the 19 studies, 16 opted to use whole blood/buffy coat/leukocytes as the source of DNA, while three studies extracted DNA from lymphocytes [28,34,35]. Except for the three studies using lymphocytes, the remaining studies, used cell populations that were a mixture of heterogeneous leukocyte subpopulations for DNA extraction. Given that leukocyte subtypes have unique DNA-methylation profiles [50,51], the associations observed in these studies could be confounded by differential leukocyte distribution. Despite blood cell composition posing a significant risk for confounding methylation measurements, almost all studies failed to adjust for this factor. Only one study [19] identifying three significantly differentially methylated markers between cases and controls reported risk estimates adjusted for leukocyte composition. While confounding introduced by leukocyte composition could be minor [52], the differences resulting from varying cell populations make the methylation measurements across studies less comparable. Nevertheless, even if differential methylation resulting from differential leukocyte distribution may be regarded as confounder from a biological perspective, it might still be useful for risk stratification based on whole blood DNA methylation patterns. 

Besides DNA source, another issue in reviewed studies is the selection of candidate genes/CpG sites to assess differential methylation that does not allow systematic and unbiased approach to risk evaluation. Most of the studies examined selected candidate genes, mainly focusing on gene promoter CpG islands and few studies focused on differentially methylated regions and body regions. Among these studies, only three studies [19,36,45] evaluated methylation at specific loci, while the rest reported methylation at the region as a whole. By utilizing primer- and probe-based assays, the studies analyzed only a few CpG dinucleotides that served as a surrogate for the methylation status of the whole region. As methylation patterns often vary largely across genomic regions and are poorly defined [53,54], the analysis of a limited number of CpG dinucleotides is less informative compared to a genome-wide analysis. However, the identification of differentially methylated regions within the selected genes provides encouraging evidence that differences in methylation at these regions should be explored further to identify specific differentially methylated loci for developing more precise risk-stratification tools.

The reviewed studies varied substantially in reporting the DNA methylation analysis results, mostly due to the lack of standardization of methodology. The studies used six types of methylation assays including variant combinations of pretreatment techniques and analytical steps to investigate DNA methylation markers. For distinguishing cancer-related aberrant methylation from baseline methylation, the use of methods that deliver quantitative methylation data is desirable. Over the last decade, technological advancements have considerably augmented the development of robust quantitative assays for analyzing DNA methylation. However, the most commonly used assays in the included studies were non- or semi-quantitative. Binary measurements of methylation obtained using MS-PCR or MS-HRM are rarely informative as thresholds used for dichotomizing the methylation data vary largely. While a few studies used quantitative methods like pyrosequencing, the method only analyzes a small range of CpG sites, and does not provide genome-wide methylation information [55,56]. Although the advantages of microarray- or sequencing-based technologies for high-throughput genome-wide methylation profiling have been effectively demonstrated [57,58], only two included [19,27] studies assessed methylation using these technologies. Potential shortcomings that may have limited the use of microarray- or sequencing-based technologies are cost considerations as well as the complexity involved in the interpretation of results. Nevertheless, in order to identify promising biomarkers for CRC risk stratification, there is an urgent need for more rigorous assessment of genome-wide methylation patterns. Such rigorous assessment involving the interrogation of methylation at over 850,000 sites across the human genome and covering 99% of RefSeq genes, is now possible using the recently introduced Illumina Infinium MethylationEPIC BeadChip [59]. In the future, more studies utilizing this technology might be warranted.

Compared to methylation of SEPT9 [22,23] in tumor-derived cell-free DNA, or to other types of molecular markers such as micro RNA [60,61], genetic [62], or proteomic markers [63,64], performance of whole-blood methylation markers for diagnosis/risk stratification of colorectal neoplasms seems much poorer. In the past 10–15 years large progress has been made in risk stratification of CRC by genetic risk variants and their combination in polygenetic risk scores [62,65,66,67,68]. Reproducible genetic risk variants have mostly been identified by hypothesis-free screening of genetic variants through genome-wide arrays in large scale international consortia including tens of thousands of CRC cases and controls, with thorough correction for multiple testing and independent validation of promising risk variants and risk scores, rather than by candidate gene approaches. It appears plausible that analogous hypothesis-free epigenome-wide methylation analyses in large scale consortia, with thorough correction for multiple testing and independent validation of promising risk variants in prospective cohorts including large screening populations will be the most promising way to go for identification of robust methylation-based risk predictors in the future. A most promising approach could then be to combine such methylation-based risk scores with polygenetic risk scores, traditional risk factor-based risk scores [69] and possibly other, yet to be established biomarker-based risk scores to come up with most informative risk prediction.

We acknowledge that there are limitations to this review. Firstly, we presented only a structured synthesis of multiple study results. Due to the heterogeneity across the reviewed studies, we did not conduct a meta-analysis combining the results of independent studies. Secondly, selection of studies may have affected our conclusions; even after developing the inclusion/exclusion criteria to ensure that all relevant studies are included, some articles could have been missed. Finally, publication bias with a tendency to publish more promising results may have led to overestimated associations in this review.

## 4. Methods

We conducted this systematic literature review according to the Preferred Reporting Items for Systematic Reviews and Meta-Analyses (PRISMA) guidelines [70].

### 4.1. Systematic Literature Search

We conducted a literature search to identify studies assessing DNA methylation in whole-blood-borne DNA from colorectal adenoma or sporadic CRC patients and healthy controls. Databases of PubMed and ISI Web of Science were searched for relevant articles until 12 November 2018. The search term combinations used were as follows: (“colorectal cancer” OR “colorectal carcinoma” OR “colorectal tumor” OR “colorectal neoplasm” OR “colorectal adenoma” OR “colon cancer” OR “colon carcinoma” OR “colon tumor” OR “colon neoplasm” OR “colon adenoma” OR “colonic cancer” OR “colonic carcinoma” OR “colonic tumor” OR “colonic neoplasm” OR “colonic adenoma” OR “rectal cancer” OR “rectal carcinoma” OR “rectal tumor” OR “rectal neoplasm” OR “cancer of colon” OR “cancer of the colon” OR “cancer of rectum” OR “cancer of the rectum” OR CRC) AND (“blood” OR “PBMC” OR “leukocyte” OR “lymphocyte”) AND (“DNA” OR “deoxyribonucleic acid” OR “ds-DNA”) AND (“methylation” OR “hypermethylation” OR “hypomethylation”) AND (“detection” OR “diagnosis” OR “screen” OR “screening” OR “marker” OR “biomarker”). Additionally, reference lists of relevant studies and reviews were scanned to identify relevant articles. Duplicate articles were removed.

### 4.2. Eligibility Criteria

We included studies if they were examining DNA methylation in whole blood samples from colorectal adenoma cases or CRC patients with sporadic disease compared to healthy individuals. Our search was restricted to human research studies in English language. The first step in the selection of eligible studies was based on reading the title and abstract. Articles were excluded if they were (1) not relevant to the topic, (2) not original articles, (3) not based on whole blood samples, (4) assessing global DNA methylation only, or (5) exclusively focusing on hereditary CRC. Then, the full texts of these articles were read and included when deemed relevant. Finally, articles that did not report any measure of relative risk or diagnostic performance or *p*-value for the comparison of methylation levels and without enough data to calculate them were also excluded. 

### 4.3. Data Extraction and Quality Assessment

Two authors (J.R.R. and Z.G.) independently extracted data from the eligible studies. Extracted variables included first author, publication year, country, study design, age of study population, DNA methylation assay, candidate gene, and relevant results including *p*-value for differential methylation levels between cases and controls, OR, and AUC. For articles not reporting the risk estimates explicitly, information was extracted from available text and tables to calculate the ORs. Discrepancies in extracted data were discussed and resolved by consensus among the authors.

The quality of included articles was assessed using the QUADAS-2 (Quality Assessment of Diagnostic Accuracy Studies 2) tool [71]. The tool was tailored to the review topic and the risk of bias and concerns regarding applicability for each study were assessed over four domains: Patient selection, index test, reference standard, and flow and timing. The risk of bias and concerns regarding applicability for each study was then rated as “high”, “low”, or “unclear”.

## 5. Conclusions

In summary, there is considerable interest in the use of whole-blood DNA methylation biomarkers to assess the likelihood of developing colorectal neoplasms. However, current risk assessment studies are inconclusive as to which methylation markers are promising for CRC risk stratification. This is due to several limitations in methodology as outlined in this article. The variation in methodology and incomplete reporting among the studies also limited the analyses of this review. It is, therefore strongly recommended that future risk assessment studies apply more standardized methods, particularly in quantifying methylation data. Although time-consuming and expensive, diagnostic and risk stratification performance should preferably be evaluated in screening cohorts or large-scale population-based cohort studies rather than case-control studies in which methylation patterns among cases may have been altered through the course of the disease, after diagnosis or even initial treatment. Integrating epigenetic and genetic markers may represent a promising approach for future CRC risk stratification schemes. Thus, further research should aim for assessment and validation of the combined performance of genetic and epigenetic markers for CRC risk prediction in order to best define the use of such signatures for research and clinical practice.

## Figures and Tables

**Table 1:** DNA methylation markers in whole blood for risk assessment of colorectal neoplasms.

Gene/LINE-1 locus	Chromosome	Gao, 2018 [37]	Zhang, 2017 ^a^ [38]	Liu, 2017 [39]	Leclerc, 2017 [42]	Heiss, 2017 [19]	Alexander, 2017 ^b^ [43]	Luo, 2016 [40]	Xiao, 2015 [41]	Ravegnini, 2015 [32]	Nüsgen, 2015 [36]	Ho, 2015 ^b^ [45]	Gao, 2012 [27]	Miroglio, 2010 [35]	Kaaks, 2009 [28]	Ally, 2009 ^a^ [33]	Ito, 2008 [34]	Ashktorab, 2007 ^b^ [44]	Miotto, 2004 [31]	Sabbioni, 2003 [30]	Report Frequency	Frequency of Significant Results
*CITED4*	1	⬆																			1	1
*GSTM2*	1												○ ^c^								1	0
*MTHFR*	1											○									1	0
*PARP1*	1												○ ^c^								1	0
*PER3*	1						⬇														1	1
*PTCH2*	1												○ ^c^								1	0
*AOX-1*	2							⬆													1	1
*CNRIP1*	2		⬆																		1	1
*L1C2*	2										○										1	0
*PER2*	2						○														1	0
*TMEFF2*	2						○													○	2	0
*ADAMTS9*	3							⬆													1	1
*MLH1*	3			○			○														2	0
*MME_*	3												○ ^c^								1	0
*RARB2*	3							○													1	0
*SEMA3F*	3												○ ^c^								1	0
*APC*	5			○			○											○			3	0
*FLT4*	5												○ ^c^								1	0
*NEUROG1*	5			⬆																	1	1
*DSP*	6												○ ^c^								1	0
*ESR1*	6						○									○ ^c^					2	0
*IRF4*	6							⬆													1	1
*L1C6*	6										○										1	0
*PLAGL1*	6												○ ^c^								1	0
*PDK4*	7				⬇																1	1
*PODXL*	7												○ ^c^								1	0
*SEMA3C*	7												○ ^c^								1	0
*SFRP4*	7						○														1	0
*SGCE*	7												○ ^c^								1	0
*CRH*	8	○																			1	0
*APBA1*	9						⬇														1	1
*CDKN2A*	9			○			○														2	0
*DAPK1*	9			⬆																	1	1
*FOXE-1*	9							○													1	0
*L1C10*	10										○										1	0
*MGMT*	10			○			○														2	0
*SFRP5*	10						○														1	0
*H19*	11													○							1	0
*IGF2*	11			⬆			○							⬇	○		○				5	2
*IL18BP*	11												○ ^c^								1	0
*KIAA1549L*	11					⬆ ^c^															1	1
*KCNK4*	11												○ ^c^								1	0
*L1C11*	11										○										1	0
*WT1*	11	⬆																			1	1
*CYP27B1*	12						○														1	0
*PDE1B_*	12												○ ^c^								1	0
*RERG*	12							⬆													1	1
*TMEM132D*	12	○																			1	0
*WIF1*	12			⬆			○														2	1
*WNT1*	12												○ ^c^								1	0
*GJB2*	13												○ ^c^								1	0
*MLH3*	14												○ ^c^								1	0
*SNRPN*	15												○ ^c^								1	0
*CDH1*	16			○																	1	0
*NDRG4*	16								○												1	0
*BRCA1*	17						○														1	0
*CA10*	17	⬆																			1	1
*HIC1*	17												○ ^c^								1	0
*NGFR*	17						○														1	0
*PER1*	17						⬇														1	1
*SEPT9*	17						○			⬇											2	1
*BCL2*	18					⬆ ^c^															1	1
*INSR*	19												○ ^c^								1	0
*CDH4*	20																		○		1	0
*DNMT3B*	20											⬆									1	1
*HCK*	20												○ ^c^								1	0
*L1C20*	20										○										1	0
*B3GALT5*	21												○ ^c^								1	0
*COL18A1*	21												○ ^c^								1	0
*TIMP3*	22						○														1	0
*DKC1*	X												○ ^c^								1	0
*GLA*	X												○ ^c^								1	0
*L1X1*	X										○										1	0
*L1X3*	X										○										1	0
*L1X4a*	X										○										1	0
*L1X5b* ^d^	X										○										1	0
*L1X6b*	X										○										1	0
*L1X8*	X										⬆ ^c^										1	1
*HBII*													○ ^c^								1	0
*MINT31*				⬆																	1	1

○ represents no difference in methylation levels between colorectal adenoma/CRC cases and controls; ⬆ represents hypermethylated marker in colorectal adenoma/CRC cases; ⬇ represents hypomethylated marker in colorectal adenoma/CRC cases. ^a^ Cases included both, colorectal adenomas and CRCs; ^b^ Cases included only colorectal adenomas; ^c^ Reported association after multiple testing correction (methylation changes of 26 CpG sites were significantly associated with CRC risk before Bonferroni correction, but none remained statistically significant after the correction); ^d^ Methylation was significantly different in female patients compared to healthy women. Abbreviations: LINE, long interspersed element.

**Table 2:** Dichotomized methylation of specific genes with significant associations to risk of colorectal neoplasms.

Gene	First Author, Year [Ref NO.]	Country	No. Cases/Controls	Age (Years) Cases/Controls	DNAm Assay	OR (95%CI)	*p*-Value *
Hypomethylation ^a^							
*PER1* *(promoter)*	Alexander, 2017 [43]	USA	38/69	--	MS-PCR	2.9 (1.1–7.7) ^b^	0.03
*APBA1* *(promoter)*	Alexander, 2017 [43]	USA	38/69	--	MS-PCR	5.3 (1.0–28.2) ^c^	0.05
*PER3* *(promoter)*	Alexander, 2017 [43]	USA	38/69	--	MS-PCR	11.1 (1.6–78.5) ^d^	0.02
Hypermethylation							
*AOX-1*	Luo, 2016 [40]	China	421/506	59.5/56.6	MS-HRM	1.72 (1.30–2.27) ^e^	0.00
*ADAMTS9*	Luo, 2016 [40]	China	421/506	59.5/56.6	MS-HRM	1.85 (1.37–2.49) ^e^	0.00
*RERG*	Luo, 2016 [40]	China	421/506	59.5/56.6	MS-HRM	2.08 (1.56–2.77) ^e^	0.00
*WIF1*	Liu, 2017 [39]	China	428/428	59.4/59.4	MS-HRM	2.44 (1.53–3.87) ^f^	<0.0001
*IGF2*	Liu, 2017 [39]	China	428/428	59.4/59.4	MS-HRM	2.54 (1.65–3.92) ^f^	<0.0001
*NEUROG1*	Liu, 2017 [39]	China	428/428	59.4/59.4	MS-HRM	2.57 (1.55–4.25) ^f^	<0.0001
*WT1*	Gao, 2018 [37]	China	466/507	60.1/56.7	MS-HRM	2.59 (1.73–3.88) ^g^	0.00
*DAPK1*	Liu, 2017 [39]	China	428/428	59.4/59.4	MS-HRM	2.95 (1.94–4.49) ^f^	<0.0001
*CITED4*	Gao, 2018 [37]	China	466/507	60.1/56.7	MS-HRM	2.96 (1.68–5.24) ^g^	0.00
*MINT31*	Liu, 2017 [39]	China	428/428	59.4/59.4	MS-HRM	4.27 (1.52–12.05) ^f^	0.01
*CA10*	Gao, 2018 [37]	China	466/507	60.1/56.7	MS-HRM	4.83 (2.82–8.28) ^g^	0.00
*IRF4*	Luo, 2016 [40]	China	421/506	59.5/56.6	MS-HRM	16.96 (5.15–55.84) ^e^	0.00

^a^ Odds of adenoma development given no methylation detected in the candidate gene. OR: ^b^ Model adjusted for vitamin C and D use and physical activity; ^c^ Model adjusted for vitamin C and D use, and ever smoking; ^d^ Model adjusted for multivitamin use, vitamin C and D use, physical activity, ever smoking, age, and being married; ^e^ Model adjusted for age, BMI, occupation and family history of cancer; ^f^ Model adjusted for age, gender, BMI, occupational physical activity, smoking, and consumption of coarse grains, fish stewed with brown sauce, fried food, leftovers. and pork; ^g^ Model adjusted for BMI, age, fruit, coarse grains, fruit can, pork intestines, fried food, garlic, and braised fish in brown sauce. *p*-value *: statistical significance for OR. Abbreviations: Ref., Reference; No., Number; DNAm, DNA methylation; OR, Odds Ratio; CI, Confidence Interval; MS-PCR, Methylation Specific Polymerase Chain Reaction; MS-HRM, Methylation-sensitive High-resolution Melting.

**Table 3:** Quantitative methylation of specific genes with significant associations to risk of colorectal neoplasms.

Gene	First Author, Year [Ref. No.]	No. Cases/ Controls	Age (Year) Cases/ Controls	DNA Methylation Assay	AUC	*p*-Value *	OR ^a^ Tertile 3 vs. 1 (95%CI)	OR ^b^ Quartile 4 vs. 1 (95%CI)	OR ^b^ per SD (95%CI)
*BCL2 (gene-body, cg12459502)*	Heiss, 2017 [19]	Screening Setting: 46/46 Clinical setting: 93/94	67/67 65/65	HM 450K MassArray	0.57 0.69	<0.05 <0.05	--	--	--
*B3GALT5* *(promoter)*	Gao, 2012 [27]	221/219	58/58	GoldenGate Cancer Panel I		0.01 ^c^	1.2 (1.06–1.37)	--	--
*COL18A1* *(promoter)*	Gao, 2012 [27]	221/219	58/58	GoldenGate Cancer Panel I		0 ^c^	1.2 (1.05–1.37)	--	--
*DKC1* *(promoter)*	Gao, 2012 [27]	221/219	58/58	GoldenGate Cancer Panel I		0.01 ^c^	1.2 (1.05–1.43)	--	--
*DNMT3B (GCA, 31351136)*	Ho, 2015 [45]	87/172	--	Sequenom EpiTYPER		0.03	--	--	1.38 (1.03–1.86)
*DNMT3B (GCA, 31351260–31351263)*	Ho, 2015 [45]	87/172	--	Sequenom EpiTYPER		0.03	--	2.07 (0.88–4.86)	--
*DSP* *(promoter)*	Gao, 2012 [27]	221/219	58/58	GoldenGate Cancer Panel I		0 ^c^	1.27 (1.12–1.43)	--	--
*FLT4* *(promoter)*	Gao, 2012 [27]	221/219	58/58	GoldenGate Cancer Panel I		0 ^c^	1.15 (0.99–1.32)	--	--
*GJB2* *(promoter)*	Gao, 2012 [27]	221/219	58/58	GoldenGate Cancer Panel I		0 ^c^	1.03 (0.9–1.18)	--	--
*GLA* *(promoter)*	Gao, 2012 [27]	221/219	58/58	GoldenGate Cancer Panel I		0 ^c^	1.45 (1.19–1.75)	--	--
*GSTM2* *(promoter)*	Gao, 2012 [27]	221/219	58/58	GoldenGate Cancer Panel I		0 ^c^	1.17 (1.03–1.34)	--	--
*HBII* *(promoter)*	Gao, 2012 [27]	221/219	58/58	GoldenGate Cancer Panel I		0 ^c^	0.87 (0.76–0.99)	--	--
*HCK* *(promoter)*	Gao, 2012 [27]	221/219	58/58	GoldenGate Cancer Panel I		0 ^c^	1.2 (1.03–1.39)	--	--
*HIC1* *(promoter)*	Gao, 2012 [27]	221/219	58/58	GoldenGate Cancer Panel I		0.01 ^c^	1.22 (1.07–1.41)	--	--
*IL18BP* *(promoter)*	Gao, 2012 [27]	221/219	58/58	GoldenGate Cancer Panel I		0.01 ^c^	1.07 (0.91–1.26)		
*INSR* *(promoter)*	Gao, 2012 [27]	221/219	58/58	GoldenGate Cancer Panel I		0 ^c^	1.24 (1.09–1.41)	--	--
*KCNK4* *(promoter)*	Gao, 2012 [27]	221/219	58/58	GoldenGate Cancer Panel I		0 ^c^	1.15 (1.01–1.31)	--	--
*KIAA1549L* *(promoter, cg04036920)*	Heiss, 2017 [19]	Screening Setting: 46/46 Clinical setting: 93/94	67/67 65/65	HM 450K MassArray	0.67 0.70	<0.05 <0.05	--	--	--
*KIAA1549L* *(promoter, cg14472551)*	Heiss, 2017 [19]	Screening Setting: 46/46 Clinical setting: 93/94	67/67 65/65	HM 450K MassArray	0.72 0.64	<0.05 <0.05	--	--	--
*L1X8 (intergenic, UBQLN2/* *Centromer)*	Nüsgen, 2015 [36]	21/59	--	Pyrosequencing	0.66	<0.05	--	--	--
*MLH3* *(promoter)*	Gao, 2012 [27]	221/219	58/58	GoldenGate Cancer Panel I		0 ^c^	1.27 (1.1–1.46)	--	--
*MME_* *(promoter)*	Gao, 2012 [27]	221/219	58/58	GoldenGate Cancer Panel I		0.01 ^c^	1.17 (1.03–1.33)	--	--
*PARP1* *(promoter)*	Gao, 2012 [27]	221/219	58/58	GoldenGate Cancer Panel I		0 ^c^	0.87 (0.76–0.99)	--	--
*PDE1B_* *(promoter)*	Gao, 2012 [27]	221/219	58/58	GoldenGate Cancer Panel I		0.01 ^c^	1.21 (1.05–1.41)	--	--
*PLAGL1* *(promoter)*	Gao, 2012 [27]	221/219	58/58	GoldenGate Cancer Panel I		0 ^c^	0.88 (0.76–1.01)	--	--
*PODXL* *(promoter)*	Gao, 2012 [27]	221/219	58/58	GoldenGate Cancer Panel I		0.01 ^c^	1.12 (0.99–1.28)	--	--
*PTCH2* *(promoter)*	Gao, 2012 [27]	221/219	58/58	GoldenGate Cancer Panel I		0 ^c^	1.26 (1.11–1.43)	--	--
*KIAA1549L* *(promoter, cg14472551)*	Heiss, 2017 [19]	Screening Setting: 46/46 Clinical setting: 93/94	67/67 65/65	HM 450K MassArray	0.72 0.64	<0.05 <0.05	--	--	--
*L1X8 (intergenic, UBQLN2/* *Centromer)*	Nüsgen, 2015 [36]	21/59	--	Pyrosequencing	0.66	<0.05	--	--	--
*MLH3* *(promoter)*	Gao, 2012 [27]	221/219	58/58	GoldenGate Cancer Panel I		0 ^c^	1.27 (1.1–1.46)	--	--
*MME_* *(promoter)*	Gao, 2012 [27]	221/219	58/58	GoldenGate Cancer Panel I		0.01 ^c^	1.17 (1.03–1.33)	--	--
*PARP1* *(promoter)*	Gao, 2012 [27]	221/219	58/58	GoldenGate Cancer Panel I		0 ^c^	0.87 (0.76–0.99)	--	--
*PDE1B_* *(promoter)*	Gao, 2012 [27]	221/219	58/58	GoldenGate Cancer Panel I		0.01 ^c^	1.21 (1.05–1.41)	--	--
*PLAGL1* *(promoter)*	Gao, 2012 [27]	221/219	58/58	GoldenGate Cancer Panel I		0 ^c^	0.88 (0.76–1.01)	--	--
*PODXL* *(promoter)*	Gao, 2012 [27]	221/219	58/58	GoldenGate Cancer Panel I		0.01 ^c^	1.12 (0.99–1.28)	--	--
*PTCH2* *(promoter)*	Gao, 2012 [27]	221/219	58/58	GoldenGate Cancer Panel I		0 ^c^	1.26 (1.11–1.43)	--	--
*SEMA3C* *(promoter)*	Gao, 2012 [27]	221/219	58/58	GoldenGate Cancer Panel I		0 ^c^	0.88 (0.77–1)	--	--
*SEMA3F* *(promoter)*	Gao, 2012 [27]	221/219	58/58	GoldenGate Cancer Panel I		0 ^c^	1.16 (1.01–1.34)	--	--
*SGCE* *(promoter)*	Gao, 2012 [27]	221/219	58/58	GoldenGate Cancer Panel I		0.01 ^c^	1.23 (1.08–1.4)	--	--
*SNRPN* *(promoter)*	Gao, 2012 [27]	221/219	58/58	GoldenGate Cancer Panel I		0.01 ^c^	0.98 (0.87–1.11)	--	--

OR: ^a^ Model adjusted for age and batch factor; ^b^ Model adjusted for sex and age. ^c^ *p*-value was significant before multiple testing correction but did not remain statistically significant after the correction. Methylation levels were adjusted for leukocyte composition and batch effects [19]. Cases included only colorectal adenomas [45]. *p*-value *: significant difference in methylation between colorectal adenoma/cancer cases and controls. Abbreviations: Ref., Reference; No., Number; OR, Odds Ratio; CI, Confidence Interval; SD, Standard Deviation; HM-450K, Human Methylation 450K; GCA, Gene-coding area.

**Table 4:** Diagnostic performance of methylation panels.

Gene Panel	First author, Year [Ref. No.]	No. Cases/Controls	Age(yrs) Cases/ Controls	DNAm Assay	AUC	Classification	OR (95%CI)	*p*- Value *
Hypomethylation								
*PER1/PER3*	Alexander, 2017 [43]	38/69	--	MS-PCR	--	--	0.50 (0.22–1.15) ^a^	0.10
*PER1/APBA1*	Alexander, 2017 [43]	38/69	--	MS-PCR	--	--	0.59 (0.26–1.33) ^a^	0.20
*PER3/APBA1*	Alexander, 2017 [43]	38/69	--	MS-PCR	--	--	0.27 (0.09–0.81) ^a^	0.02
*PER1/PER3/APBA1*	Alexander, 2017 [43]	38/69	--	MS-PCR	--	--	0.57 (0.25–1.27) ^a^	0.17
Hypermethylation								
*MCSM* *(WT1, CA10, CITED4 & TMEM132D)*	Gao, 2018 [37]	466/507	60.1/56.7	MS-HRM	--	Non-MCSM	1.00 ^b^	
						(no methylated gene) (ref)		
						MCSM-L (≤2 methylated genes)	1.43 (0.50–4.05)	0.50
						MCSM-H (≥3 methylated genes)	4.32 (1.53–12.2)	0.01
						MCSM	2.53 (0.92–6.94)	0.07
*MRS_10* *(APC+CDH1+* *CDKN2A+DAPK1+* *IGF2+MGMT+* *MINT31+MLH1+* *NEUROG1+WIF1)*	Liu, 2017 [39]	428/428	59.4/59.4	MS-HRM	0.69 (0.66–0.73), *p* < 0.0001	Low predicted probability ≤0.5 (ref)	1.00 ^c^	
						Medium	3.85 (2.72–5.45)	<0.0001
						(0.5 < predicted probability ≤ 0.7)		
						High	6.51 (3.77–11.27)	<0.0001
						(predicted probability >0.7)		
						Medium or High	4.39 (3.19–6.05)	<0.0001
*Markers-only model (cg04036920, cg14472551 & cg12459502)*	Heiss, 2017 [19]	SS: 46/46 CS: 93/94	67/67 65/65	HM 450K	0.69 (0.55, 0.82) ^d^ 0.73 (0.63, 0.83) ^d^			
*Full model (combining sex, age, cg04036920, cg14472551 & cg12459502)*	Heiss, 2017 [19]	SS: 46/46 CS: 93/94	67/67 65/65	HM 450K	0.69 (0.54, 0.83) ^d^ 0.73 (0.61, 0.82) ^d^			
*MCSM* *(AOX-1, RARB2, RERG, ADAMTS9, IRF4, & FOXE-1)*	Luo, 2016 [40]	421/506	59.5/56.6	MS-HRM	--	Non-MCSM	1.00 ^e^	
						(no methylated gene) (ref)		
						MCSM-L (1 methylated gene)	1.23 (0.87–1.75)	0.24
						MCSM-H (≥2 methylated genes	1.79 (1.28–2.52)	0.00
						(except for RARB2))		
						MCSM	1.50 (1.11–2.03)	0.01

^a^ Crude OR (95% CI) for dichotomized methylation of gene panel and adenoma risk; coding of methylation status seems erroneous and could not be clarified. ^b^ ORs adjusted for BMI, age, fruit, coarse grains, fruit can, pork intestines, fried food, garlic, and braised fish in brown sauce. ^c^ ORs adjusted for age, gender, BMI, occupational physical activity, smoking, and consumption of coarse grains, fish stewed with brown sauce, fried food, leftovers, and pork. ^d^ Methylation levels were adjusted for leukocyte composition and batch effects. ^e^ Model adjusted for age, BMI, occupation and family history of cancer. *p*-value *: statistical significance for OR. Abbreviations: Ref., Reference; No., Number; DNAm, DNA methylation; OR, Odds Ratio; CI, Confidence Interval; SS, Screening Setting; CS, Clinical setting; MRS, methylation risk score; MCSM, multiple CpG site methylation; HM-450K., Human Methylation 450k; MS-PCR, Methylation Specific Polymerase Chain Reaction; MS-HRM, Methylation-sensitive High-resolution Melting.

## Supplementary Materials

The following are available online at https://www.mdpi.com/2072-6694/11/7/912/s1, Figure S1: QUADAS overview, Figure S2: QUADAS-2 risk of bias assessment of reviewed studies, Table S1: Study characteristics of reviewed studies, Table S2: DNA methylation difference between colorectal adenoma/CRC cases and controls reported by *p*-value only, Table S3: Methylation of genes with no significant association to colorectal adenoma/CRC risk.

## References

[1] Bray F., Ferlay J., Soerjomataram I., Siegel R.L., Torre L.A., Jemal A. (2018). Global cancer statistics 2018: Globocan estimates of incidence and mortality worldwide for 36 cancers in 185 countries. CA Cancer J. Clin..

[2] Brenner H., Stock C., Hoffmeister M. (2014). Effect of screening sigmoidoscopy and screening colonoscopy on colorectal cancer incidence and mortality: Systematic review and meta-analysis of randomised controlled trials and observational studies. BMJ.

[3] Nishihara R., Wu K., Lochhead P., Morikawa T., Liao X., Qian Z.R., Inamura K., Kim S.A., Kuchiba A., Yamauchi M. (2013). Long-term colorectal-cancer incidence and mortality after lower endoscopy. New Engl. J. Med..

[4] Shaukat A., Mongin S.J., Geisser M.S., Lederle F.A., Bond J.H., Mandel J.S., Church T.R. (2013). Long-term mortality after screening for colorectal cancer. New Engl. J. Med..

[5] Khalid-de Bakker C., Jonkers D., Smits K., Mesters I., Masclee A., Stockbrugger R. (2011). Participation in colorectal cancer screening trials after first-time invitation: A systematic review. Endoscopy.

[6] Sabatino S.A., White M.C., Thompson T.D., Klabunde C.N. (2015). Cancer screening test use—United States, 2013. Morb. Mortal. Wkly. Rep..

[7] Klabunde C.N., Vernon S.W., Nadel M.R., Breen N., Seeff L.C., Brown M.L. (2005). Barriers to colorectal cancer screening: A comparison of reports from primary care physicians and average-risk adults. Med Care.

[8] Jones R.M., Devers K.J., Kuzel A.J., Woolf S.H. (2010). Patient-reported barriers to colorectal cancer screening: A mixed-methods analysis. Am. J. Prev. Med..

[9] Muliira J.K., D’Souza M.S., Ahmed S.M., Al-Dhahli S.N., Al-Jahwari F.R. (2016). Barriers to colorectal cancer screening in primary care settings: Attitudes and knowledge of nurses and physicians. Asia-Pac. J. Oncol. Nurs..

[10] Schroy P.C., Duhovic E., Chen C.A., Heeren T.C., Lopez W., Apodaca D.L., Wong J.B. (2016). Risk stratification and shared decision making for colorectal cancer screening: A randomized controlled trial. Med Decis. Mak. Int. J. Soc. Med Decis. Mak..

[11] Pancione M., Remo A., Colantuoni V. (2012). Genetic and epigenetic events generate multiple pathways in colorectal cancer progression. Pathol. Res. Int..

[12] Chan A.O., Broaddus R.R., Houlihan P.S., Issa J.P., Hamilton S.R., Rashid A. (2002). Cpg island methylation in aberrant crypt foci of the colorectum. Am. J. Pathol..

[13] Kim Y.H., Petko Z., Dzieciatkowski S., Lin L., Ghiassi M., Stain S., Chapman W.C., Washington M.K., Willis J., Markowitz S.D. (2006). Cpg island methylation of genes accumulates during the adenoma progression step of the multistep pathogenesis of colorectal cancer. Genes Chromosomes Cancer.

[14] Rashid A., Shen L., Morris J.S., Issa J.P., Hamilton S.R. (2001). Cpg island methylation in colorectal adenomas. Am. J. Pathol..

[15] Ahlquist T., Lind G.E., Costa V.L., Meling G.I., Vatn M., Hoff G.S., Rognum T.O., Skotheim R.I., Thiis-Evensen E., Lothe R.A. (2008). Gene methylation profiles of normal mucosa, and benign and malignant colorectal tumors identify early onset markers. Mol. Cancer.

[16] Azuara D., Rodriguez-Moranta F., de Oca J., Soriano-Izquierdo A., Mora J., Guardiola J., Biondo S., Blanco I., Peinado M.A., Moreno V. (2010). Novel methylation panel for the early detection of colorectal tumors in stool DNA. Clin. Colorectal Cancer.

[17] Cai Q., Gao Y.T., Chow W.H., Shu X.O., Yang G., Ji B.T., Wen W., Rothman N., Li H.L., Morrow J.D. (2006). Prospective study of urinary prostaglandin e2 metabolite and colorectal cancer risk. J. Clin. Oncol. Off. J. Am. Soc. Clin. Oncol..

[18] Harada T., Yamamoto E., Yamano H.O., Nojima M., Maruyama R., Kumegawa K., Ashida M., Yoshikawa K., Kimura T., Harada E. (2014). Analysis of DNA methylation in bowel lavage fluid for detection of colorectal cancer. Cancer Prev. Res..

[19] Heiss J.A., Brenner H. (2017). Epigenome-wide discovery and evaluation of leukocyte DNA methylation markers for the detection of colorectal cancer in a screening setting. Clin. Epigenet..

[20] Adler A., Geiger S., Keil A., Bias H., Schatz P., deVos T., Dhein J., Zimmermann M., Tauber R., Wiedenmann B. (2014). Improving compliance to colorectal cancer screening using blood and stool based tests in patients refusing screening colonoscopy in germany. BMC Gastroenterol..

[21] Liles E.G., Coronado G.D., Perrin N., Harte A.H., Nungesser R., Quigley N., Potter N.T., Weiss G., Koenig T., deVos T. (2017). Uptake of a colorectal cancer screening blood test is higher than of a fecal test offered in clinic: A randomized trial. Cancer Treat. Res. Commun..

[22] Bergheim J., Semaan A., Gevensleben H., Groening S., Knoblich A., Dietrich J., Weber J., Kalff J.C., Bootz F., Kristiansen G. (2018). Potential of quantitative sept9 and shox2 methylation in plasmatic circulating cell-free DNA as auxiliary staging parameter in colorectal cancer: A prospective observational cohort study. Br. J. Cancer.

[23] Xie L., Jiang X., Li Q., Sun Z., Quan W., Duan Y., Li D., Chen T. (2018). Diagnostic value of methylated septin9 for colorectal cancer detection. Front. Oncol..

[24] Gormally E., Caboux E., Vineis P., Hainaut P. (2007). Circulating free DNA in plasma or serum as biomarker of carcinogenesis: Practical aspects and biological significance. Mutat. Res..

[25] Li L., Choi J.Y., Lee K.M., Sung H., Park S.K., Oze I., Pan K.F., You W.C., Chen Y.X., Fang J.Y. (2012). DNA methylation in peripheral blood: A potential biomarker for cancer molecular epidemiology. J. Epidemiol..

[26] Page K., Guttery D.S., Zahra N., Primrose L., Elshaw S.R., Pringle J.H., Blighe K., Marchese S.D., Hills A., Woodley L. (2013). Influence of plasma processing on recovery and analysis of circulating nucleic acids. PLoS ONE.

[27] Gao Y., Killian K., Zhang H., Yu K., Li Q.Z., Weinstein S., Virtamo J., Tucker M., Taylor P., Albanes D. (2012). Leukocyte DNA methylation and colorectal cancer among male smokers. World J. Gastrointest. Oncol..

[28] Kaaks R., Stattin P., Villar S., Poetsch A.R., Dossus L., Nieters A., Riboli E., Palmqvist R., Hallmans G., Plass C. (2009). Insulin-like growth factor-ii methylation status in lymphocyte DNA and colon cancer risk in the northern sweden health and disease cohort. Cancer Res..

[29] Marsit C., Christensen B. (2013). Blood-derived DNA methylation markers of cancer risk. Adv. Exp. Med. Biol..

[30] Sabbioni S., Miotto E., Veronese A., Sattin E., Gramantieri L., Bolondi L., Calin G.A., Gafa R., Lanza G., Carli G. (2003). Multigene methylation analysis of gastrointestinal tumors: Tpef emerges as a frequent tumor-specific aberrantly methylated marker that can be detected in peripheral blood. Mol. Diagn. J. Devoted Underst. Hum. Dis. Clin. Appl. Mol. Biol..

[31] Miotto E., Sabbioni S., Veronese A., Calin G.A., Gullini S., Liboni A., Gramantieri L., Bolondi L., Ferrazzi E., Gafa R. (2004). Frequent aberrant methylation of the cdh4 gene promoter in human colorectal and gastric cancer. Cancer Res..

[32] Ravegnini G., Zolezzi Moraga J.M., Maffei F., Musti M., Zenesini C., Simeon V., Sammarini G., Festi D., Hrelia P., Angelini S. (2015). Simultaneous analysis of sept9 promoter methylation status, micronuclei frequency, and folate-related gene polymorphisms: The potential for a novel blood-based colorectal cancer biomarker. Int. J. Mol. Sci..

[33] Ally M.S., Al-Ghnaniem R., Pufulete M. (2009). The relationship between gene-specific DNA methylation in leukocytes and normal colorectal mucosa in subjects with and without colorectal tumors. Cancer Epidemiol. Biomark. Prev. Publ. Am. Assoc. Cancer Res. Cosponsored Am. Soc. Prev. Oncol..

[34] Ito Y., Koessler T., Ibrahim A.E., Rai S., Vowler S.L., Abu-Amero S., Silva A.L., Maia A.T., Huddleston J.E., Uribe-Lewis S. (2008). Somatically acquired hypomethylation of igf2 in breast and colorectal cancer. Hum. Mol. Genet..

[35] Miroglio A., Jammes H., Tost J., Ponger L., Gut I.G., El Abdalaoui H., Coste J., Chaussade S., Arimondo P.B., Lamarque D. (2010). Specific hypomethylated cpgs at the igf2 locus act as an epigenetic biomarker for familial adenomatous polyposis colorectal cancer. Epigenomics.

[36] Nüsgen N., Goering W., Dauksa A., Biswas A., Jamil M.A., Dimitriou I., Sharma A., Singer H., Fimmers R., Fröhlich H. (2015). Inter-locus as well as intra-locus heterogeneity in line-1 promoter methylation in common human cancers suggests selective demethylation pressure at specific cpgs. Clin. Epigenet..

[37] Gao H.L., Wang X., Sun H.R., Zhou J.D., Lin S.Q., Xing Y.H., Zhu L., Zhou H.B., Zhao Y.S., Chi Q. (2018). Methylation status of transcriptional modulatory genes associated with colorectal cancer in northeast china. Gut Liver.

[38] Zhang T., Cui G., Yao Y.L., Wang Q.C., Gu H.G., Li X.N., Zhang H., Feng W.M., Shi Q.L., Cui W.W. (2017). Value of cnrip1 promoter methylation in colorectal cancer screening and prognosis assessment and its influence on the activity of cancer cells. Arch. Med Sci..

[39] Liu Y., Wang Y., Hu F., Sun H., Zhang Z., Wang X., Luo X., Zhu L., Huang R., Li Y. (2017). Multiple gene-specific DNA methylation in blood leukocytes and colorectal cancer risk: A case-control study in china. Oncotarget.

[40] Luo X., Huang R., Sun H., Liu Y., Bi H., Li J., Yu H., Sun J., Lin S., Cui B. (2016). Methylation of a panel of genes in peripheral blood leukocytes is associated with colorectal cancer. Sci. Rep..

[41] Xiao W., Zhao H., Dong W., Li Q., Zhu J., Li G., Zhang S., Ye M. (2015). Quantitative detection of methylated ndrg4 gene as a candidate biomarker for diagnosis of colorectal cancer. Oncol. Lett..

[42] Leclerc D., Pham D.N., Levesque N., Truongcao M., Foulkes W.D., Sapienza C., Rozen R. (2017). Oncogenic role of pdk4 in human colon cancer cells. Br. J. Cancer.

[43] Alexander M., Burch J.B., Steck S.E., Chen C.F., Hurley T.G., Cavicchia P., Shivappa N., Guess J., Zhang H., Youngstedt S.D. (2017). Case-control study of candidate gene methylation and adenomatous polyp formation. Int. J. Colorectal Dis..

[44] Ashktorab H., Begum R., Akhgar A., Smoot D.T., Elbedawi M., Daremipouran M., Zhao A., Momen B., Giardiello F.M. (2007). Folate status and risk of colorectal polyps in african americans. Dig. Dis. Sci..

[45] Ho V., Ashbury J.E., Taylor S., Vanner S., King W.D. (2015). Gene-specific DNA methylation of dnmt3b and mthfr and colorectal adenoma risk. Mutat. Res..

[46] Bretthauer M., Kaminski M.F., Loberg M., Zauber A.G., Regula J., Kuipers E.J., Hernan M.A., McFadden E., Sunde A., Kalager M. (2016). Population-based colonoscopy screening for colorectal cancer: A randomized clinical trial. JAMA Intern. Med..

[47] Oines M., Helsingen L.M., Bretthauer M., Emilsson L. (2017). Epidemiology and risk factors of colorectal polyps. Best Pract. Res. Clin. Gastroenterol..

[48] Fraser H.B., Lam L.L., Neumann S.M., Kobor M.S. (2012). Population-specificity of human DNA methylation. Genome Biol..

[49] Xia Y.Y., Ding Y.B., Liu X.Q., Chen X.M., Cheng S.Q., Li L.B., Ma M.F., He J.L., Wang Y.X. (2014). Racial/ethnic disparities in human DNA methylation. Biochim. Biophys. Acta.

[50] Reinius L.E., Acevedo N., Joerink M., Pershagen G., Dahlen S.E., Greco D., Soderhall C., Scheynius A., Kere J. (2012). Differential DNA methylation in purified human blood cells: Implications for cell lineage and studies on disease susceptibility. PloS ONE.

[51] Terry M.B., Delgado-Cruzata L., Vin-Raviv N., Wu H.C., Santella R.M. (2011). DNA methylation in white blood cells: Association with risk factors in epidemiologic studies. Epigenetics.

[52] Heiss J.A., Brenner H. (2017). Impact of confounding by leukocyte composition on associations of leukocyte DNA methylation with common risk factors. Epigenomics.

[53] Mikeska T., Candiloro I.L., Dobrovic A. (2010). The implications of heterogeneous DNA methylation for the accurate quantification of methylation. Epigenomics.

[54] Ushijima T. (2005). Detection and interpretation of altered methylation patterns in cancer cells. Nat. Rev. Cancer.

[55] Delaney C., Garg S.K., Yung R. (2015). Analysis of DNA methylation by pyrosequencing. Methods Mol. Biol..

[56] Tost J., Gut I.G. (2007). DNA methylation analysis by pyrosequencing. Nat. Protoc..

[57] Huang Y.W., Huang T.H., Wang L.S. (2010). Profiling DNA methylomes from microarray to genome-scale sequencing. Technol. Cancer Res. Treat..

[58] Yong W.S., Hsu F.M., Chen P.Y. (2016). Profiling genome-wide DNA methylation. Epigenet. Chromatin.

[59] Pidsley R., Zotenko E., Peters T.J., Lawrence M.G., Risbridger G.P., Molloy P., Van Djik S., Muhlhausler B., Stirzaker C., Clark S.J. (2016). Critical evaluation of the illumina methylationepic beadchip microarray for whole-genome DNA methylation profiling. Genome Biol..

[60] Herreros-Villanueva M., Duran-Sanchon S., Martin A.C., Perez-Palacios R., Vila-Navarro E., Marcuello M., Diaz-Centeno M., Cubiella J., Diez M.S., Bujanda L. (2019). Plasma microrna signature validation for early detection of colorectal cancer. Clin. Transl. Gastroenterol..

[61] Toiyama Y., Takahashi M., Hur K., Nagasaka T., Tanaka K., Inoue Y., Kusunoki M., Boland C.R., Goel A. (2013). Serum mir-21 as a diagnostic and prognostic biomarker in colorectal cancer. J. Natl. Cancer Inst..

[62] Weigl K., Thomsen H., Balavarca Y., Hellwege J.N., Shrubsole M.J., Brenner H. (2018). Genetic risk score is associated with prevalence of advanced neoplasms in a colorectal cancer screening population. Gastroenterology.

[63] Chen H., Qian J., Werner S., Cuk K., Knebel P., Brenner H. (2017). Development and validation of a panel of five proteins as blood biomarkers for early detection of colorectal cancer. Clin. Epidemiol..

[64] Chen H., Zucknick M., Werner S., Knebel P., Brenner H. (2015). Head-to-head comparison and evaluation of 92 plasma protein biomarkers for early detection of colorectal cancer in a true screening setting. Clin. Cancer Res. Off. J. Am. Assoc. Cancer Res..

[65] Balavarca Y., Weigl K., Thomsen H., Brenner H. (2019). Performance of individual and joint risk stratification by an environmental risk score and a genetic risk score in a colorectal cancer screening setting. Int. J. Cancer.

[66] Hsu L., Jeon J., Brenner H., Gruber S.B., Schoen R.E., Berndt S.I., Chan A.T., Chang-Claude J., Du M., Gong J. (2015). A model to determine colorectal cancer risk using common genetic susceptibility loci. Gastroenterology.

[67] Jeon J., Du M., Schoen R.E., Hoffmeister M., Newcomb P.A., Berndt S.I., Caan B., Campbell P.T., Chan A.T., Chang-Claude J. (2018). Determining risk of colorectal cancer and starting age of screening based on lifestyle, environmental, and genetic factors. Gastroenterology.

[68] Schumacher F.R., Schmit S.L., Jiao S., Edlund C.K., Wang H., Zhang B., Hsu L., Huang S.C., Fischer C.P., Harju J.F. (2015). Genome-wide association study of colorectal cancer identifies six new susceptibility loci. Nat. Commun..

[69] Peng L., Weigl K., Boakye D., Brenner H. (2018). Risk scores for predicting advanced colorectal neoplasia in the average-risk population: A systematic review and meta-analysis. Am. J. Gastroenterol..

[70] Moher D., Liberati A., Tetzlaff J., Altman D.G. (2010). Preferred reporting items for systematic reviews and meta-analyses: The prisma statement. Int. J. Surg..

[71] Whiting P.F., Rutjes A.W., Westwood M.E., Mallett S., Deeks J.J., Reitsma J.B., Leeflang M.M., Sterne J.A., Bossuyt P.M. (2011). Quadas-2: A revised tool for the quality assessment of diagnostic accuracy studies. Ann. Intern. Med..

