# Supplementary material for: Whole-blood DNA Methylation Markers for Risk Stratification in Colorectal Cancer Screening: A Systematic Review
Source: Cancers (Basel). 2019 Jun 28;11(7):912. doi: 10.3390/cancers11070912 (PMC6678372; doi:10.3390/cancers11070912)
Supplement: Supplementary file 1 [file cancers-11-00912-s001.pdf]

*Supplementary Materials*

# Whole-blood DNA Methylation Markers for Risk Stratification in Colorectal Cancer Screening: A Systematic Review

**Janhavi R. Raut, Zhong Guan, Petra Schrotz-King and Hermann Brenner**

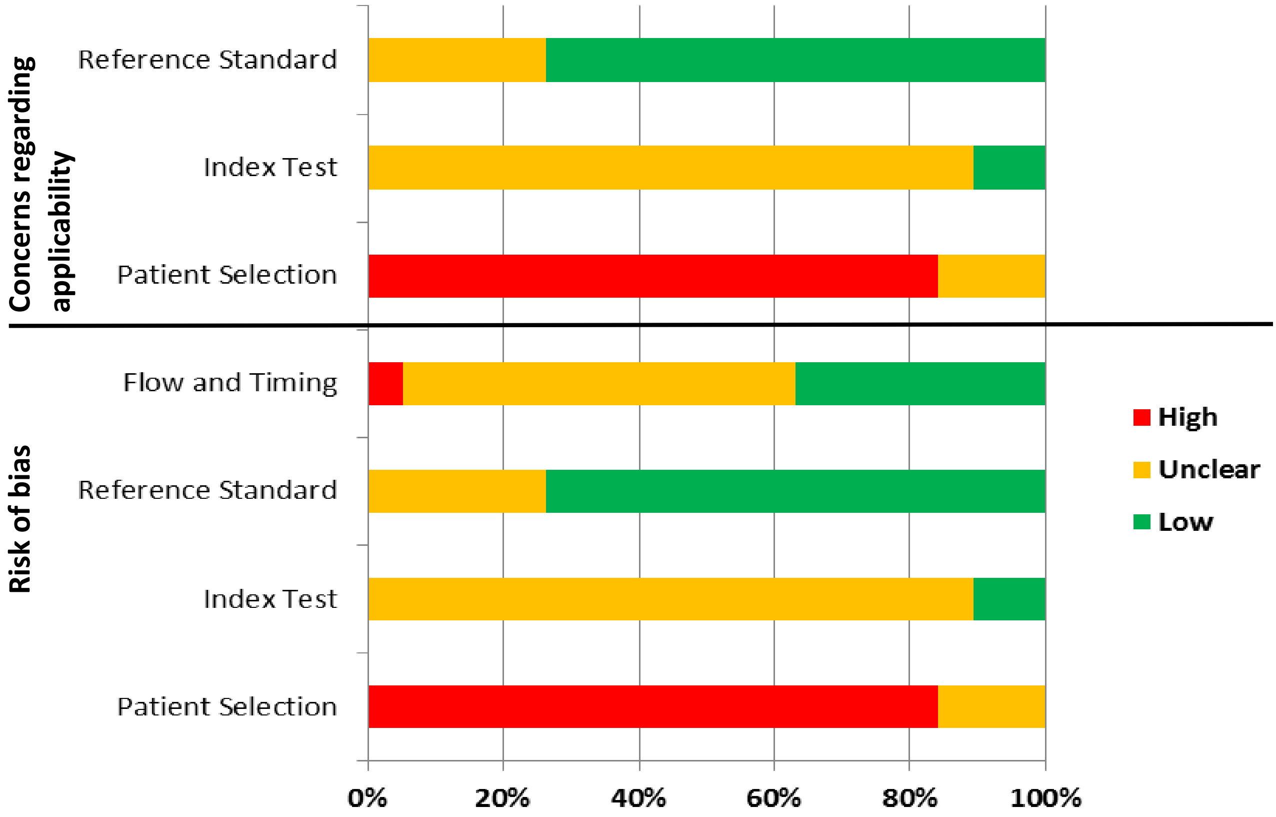


**Figure S1.** QUADAS Overview. Note: red bar, proportion with high risk; green bar, proportion with low risk; yellow bar, proportion with unclear risk.

| | Risk of Bias | | | | Applicablity Concerns | | |
|---|---|---|---|---|---|---|---|
| | Patient Selection | Index Test | Reference Standard | Flow and Timing | Patient Selection | Index Test | Reference Standard |
| Gao, 2018 [37] | High | Unclear | Low | Unclear | High | Unclear | Low |
| Zhang, 2017 [38] | High | Unclear | Low | Low | High | Unclear | Low |
| Liu, 2017 [39] | High | Low | Low | Unclear | High | Low | Low |
| Leclerc, 2017 [42] | High | Unclear | Unclear | Unclear | High | Unclear | Unclear |
| Heiss, 2017 [19] | Unclear | Low | Low | Low | Unclear | Low | Low |
| Alexander, 2017 [43] | High | Unclear | Low | Low | High | Unclear | Low |
| Luo, 2016 [40] | High | Unclear | Low | High | High | Unclear | Low |
| Xiao, 2015 [41] | High | Unclear | Low | Unclear | High | Unclear | Low |
| Ravegnini, 2015 [32] | High | Unclear | Low | Low | High | Unclear | Low |
| Nüsgen, 2015 [36] | High | Unclear | Low | Low | High | Unclear | Low |
| Ho, 2015 [45] | High | Unclear | Low | Low | High | Unclear | Low |
| Gao, 2012 [27] | High | Unclear | Unclear | Unclear | High | Unclear | Unclear |
| Miroglio, 2010 [35] | High | Unclear | Unclear | Unclear | High | Unclear | Unclear |
| Kaaks, 2009 [28] | High | Unclear | Low | Unclear | High | Unclear | Low |
| Ally, 2009 [33] | High | Unclear | Low | Low | High | Unclear | Low |
| Ito, 2008 [34] | Unclear | Unclear | Unclear | Unclear | Unclear | Unclear | Unclear |
| Ashktorab, 2007 [44] | High | Unclear | Low | Unclear | High | Unclear | Low |
| Miotto, 2004 [31] | Unclear | Unclear | Low | Unclear | Unclear | Unclear | Low |
| Sabbioni, 2003 [30] | High | Unclear | Unclear | Unclear | High | Unclear | Unclear |

**High** **Unclear** **Low**

**Figure S2.** QUADAS-2 risk of bias assessment of reviewed studies [71].

**Supplementary Table S1.** Study characteristics of reviewed studies.

| First author, Year [Ref. No.] | Study Design, Country | Study Group | Ascertainment of Cn by colonoscopy/ histopathology | No. | Mean Age (years) | DNAm assay | Reported gene/ LINE-1 region |
|---|---|---|---|---|---|---|---|
| Gao, 2018 [37] | Case-control<br>China | CRC<br>Cn (non-gastrointestinal disease subjects) | Unclear | 466<br>507 | 60.1<br>56.7 | MS-HRM | *CA10 (-)*<br>*CRH (-)*<br>*TMEM132D (-)*<br>*CITED4 (-)*<br>*WT1 (-)* |
| Liu, 2017 [39] | Case-control<br>China | CRC<br>Cn (Cancer-free subjects) | Unclear | 428<br>428 | 59.4<br>59.4 | MS-HRM | *APC (-)*<br>*CDH1 (-)*<br>*CDKN2A (-)*<br>*DAPK1 (-)*<br>*IGF2 (-)*<br>*MGMT (-)*<br>*MINT31 (-)*<br>*MLH1 (-)*<br>*NEUROG1 (-)*<br>*WIF1 (-)* |
| Leclerc, 2017 [42] | Case-control<br>USA | Cohort 1: CRC<br>Cn (Polyps/ Polyp-free)<br>Cohort 2: CRC<br>Cn (Cancer-free subjects) | Yes<br><br>Unclear | 40<br>40<br>18<br>29 | 59<br>58<br>60<br>60 | Pyrosequencing | *PDK4 (-)* |

**Supplementary Table S1.** *Cont.*

| First author, Year [Ref. No.] | Study Design, Country | Study Group | Ascertainment of Cn by colonoscopy/ histopathology | No. | Mean Age (years) | DNAm assay | Reported gene/ LINE-1 region |
|---|---|---|---|---|---|---|---|
| Heiss, 2017 [19] | Case-control Germany | Screening Setting: CRC | Yes | 46 | 67 | HM 450K MassArray | *KIAA1549L (promoter, cg04036920 & cg14472551)* *BCL2 (body, cg12459502)* |
| | | Cn (HPP/ normal colonic mucosa) | | 46 | 67 | | |
| | | Clinical setting: CRC | Unclear | 93 | 65 | | |
| | | Cn (from population registries) | | 94 | 65 | | |
| Alexander, 2017 [43] | Case-control USA | Adenoma (non-advanced) | Yes | 38 | - | MS-PCR | *APC (promoter)* *BRCA1 (promoter)* *APBA1 (promoter)* *PER1 (promoter)* *PER3 (promoter)* *SFRP4 (promoter)* *SFRP5 (promoter)* *TIMP3 (promoter)* *TMEFF2 (promoter)* *WIF1 (promoter)* *CDKN2A (promoter)* *CYP27B1 (promoter)* *ESR1 (promoter)* *IGF2 (promoter)* *MGMT (promoter)* *MLH1 (promoter)* *NGFR (promoter)* *PER2 (promoter)* |
| | | Cn (HPP/ normal colonic mucosa) | | 69 | | | |

**Supplementary Table S1.** *Cont.*

| First author, Year [Ref. No.] | Study Design, Country | Study Group | Ascertainment of Cn by colonoscopy/ histopathology | No. | Mean Age (years) | DNAm assay | Reported gene/ LINE-1 region |
|---|---|---|---|---|---|---|---|
| Alexander, 2017 [43] | Case-control USA | Adenoma (non-advanced)<br>Cn (HPP/ normal colonic mucosa) | Yes | 38<br>69 | - | MS-PCR | *SEPT9 (promoter)* |
| Zhang, 2017 [38] | Case-control China | Adenoma<br>CRC<br>Cn (healthy subjects) | Yes | 20<br>50<br>20 | 52.6<br>69.4<br>49.2 | qMS-PCR, Pyrosequencing | *CNRIP1 (promoter)* |
| Luo, 2016 [40] | Case-control China | CRC<br>Cn (non-gastrointestinal disease subjects) | Unclear | 421<br>506 | 59.5<br>56.6 | MS-HRM | *IRF4 (-)*<br>*FOXE-1 (-)*<br>*AOX-1 (-)*<br>*ADAMTS9 (-)*<br>*RERG (-)*<br>*RARB2 (-)* |
| Xiao, 2015 [41] | Case-control China | CRC<br>Cn (healthy subjects) | Unclear | 84<br>16 | 56 median<br>55.6 median | n-MS-PCR | *NDRG4 (promoter)* |
| Ravegnini, 2015 [72] | Case-control Italy | CRC<br>Cn (normal colonic mucosa) | Yes | 27<br>26 | 67.5<br>59.6 | MS-PCR | *SEPT9 (promoter)* |
| Nüsgen, 2015 [36] | Case-control Lithuania | CRC<br>Cn (healthy subjects) | Unclear | 21<br>59 | -- | Pyrosequencing | *LINE-1 loci: L1C2*<br>*L1C6*<br>*L1C10*<br>*L1C11*<br>*L1C20*<br>*L1X1*<br>*L1X3* |

**Supplementary Table S1.** *Cont.*

| First author, Year [Ref. No.] | Study Design, Country | Study Group | Ascertainment of Cn by colonoscopy/ histopathology | No. | Mean Age (years) | DNAm assay | Reported gene/ LINE-1 region |
|---|---|---|---|---|---|---|---|
| Nüsgen, 2015 [36] | Case-control Lithuania | CRC<br>Cn (healthy subjects) | Unclear | 21<br>59 | -- | Pyrosequencing | *L1X4a*<br>*L1X5b*<br>*L1X6b*<br>*L1X8* |
| Ho, 2015 [45] | Case-control Canada | Adenoma (TA/ VA/ TVA)<br>Cn (normal colonic mucosa) | Yes | 181<br>88 | -- | Sequenom EpiTYPER | *MTHFR (28 CpG sites, TSS & Gene-coding area) DNMT3B (66 CpG sites, TSS & Gene-coding area including 31350991-31350995, 31351072, 31351122, 31351136, 31351144, 31351172-31351174, 31351216, 31351226-31351230 & 31351260–31351263)* |
| Gao, 2012 [27] | NCC Finland | CRC<br>Cn (Cancer-free subjects) | Unclear | 221<br>219 | 58<br>58 | Illumina GoldenGate Methylation Cancer Panel I | *DSP_P440_R*<br>*MLH3*<br>*FLT4*<br>*INSR*<br>*GLA*<br>*PTCH2*<br>*GSTM2*<br>*HCK*<br>*PLAGL1*<br>*PARP1* |

**Supplementary Table S1.** *Cont.*

| First author, Year [Ref. No.] | Study Design, Country | Study Group | Ascertainment of Cn by colonoscopy/ histopathology | No. | Mean Age (years) | DNAm assay | Reported gene/ LINE-1 region |
|---|---|---|---|---|---|---|---|
| Gao, 2012 [27] | NCC<br>Finland | CRC<br>Cn (Cancer-free subjects) | Unclear | 221<br>219 | 58<br>58 | Illumina GoldenGate Methylation Cancer Panel I | *COL18A1*<br>*KCNK4*<br>*GJB2*<br>*SEMA3F*<br>*HBII*<br>*SEMA3C*<br>*MME_*<br>*WNT1_*<br>*SGCE*<br>*PDE1B_*<br>*HIC1*<br>*PODXL*<br>*IL18BP*<br>*DKC1*<br>*B3GALT5*<br>*SNRPN* |
| Miroglio, 2010 [35] | Case-control<br>France | CRC<br>Cn (healthy subjects) | Unclear | 26<br>19 | 64<br>40 | Pyrosequencing | *IGF2 (DMR0 between 2nd & 3rd exons)*<br>*IGF2 (DMR2 between 8th & 9th exons)*<br>*H19 DMR (4-kb upstream of the TSS)* |

**Supplementary Table S1.** *Cont.*

| First author, Year [Ref. No.] | Study Design, Country | Study Group | Ascertainment of Cn by colonoscopy/ histopathology | No. | Mean Age (years) | DNAm assay | Reported gene/ LINE-1 region |
|---|---|---|---|---|---|---|---|
| Kaaks, 2009 [28] | NCC<br>Sweden | CRC<br>Cn (Alive and Cancer-free subjects) | Unclear | 97<br>190 | 58<br>58 | SOMA | *IGF2 (DMR in 2nd intron)* |
| Ally, 2009 [33] | Case-control<br>UK | Adenoma (≥1 adenoma; TA/ VA/ TVA/ SA)<br>CRC<br>Cn (normal colonic mucosa) | Yes | 30<br>27<br>57 | 65.5<br>68.4<br>57.5 | Pyrosequencing | *ESR1(promoter & 1st exon)* |
| Ito, 2008 [34] | NCC<br>UK<br>CS<br>UK | Cohort 1: CRC<br>Cn<br>Cohort 2: CRC<br>Cn | Unclear | 225<br>435<br>188<br>94 | 66.1<br>66.1<br>--<br>-- | Pyrosequencing | *IGF2 (DMR0 promoter proximal sequence CpG12-17)* |
| Ashktorab, 2007 [44] | Case-control<br>USA | Adenomatous polyp/ HPP<br>Cn (Polyp-free) | Yes | 19<br>29 | 60.5<br>63.5 | MS-PCR | *APC (promoter)* |
| Miotto, 2004 [31] | Case-control<br>Italy | CRC<br>Cn (healthy subjects) | Unclear | 46<br>17 | -- | Semi n-MS-PCR | *CDH4 (promoter)* |
| Sabbioni, 2003 [30] | Case-control<br>Italy | CRC<br>Cn (healthy subjects) | Unclear | 29<br>16 | - | MS-PCR | *TMEFF2* |

Abbreviations: Ref., Reference; No., Number; NCC., Nested Case control (nested within a prospective cohort study); CRC, colorectal cancer; Cn, controls; DNAm, DNA methylation; HPP, hyperplastic polyp; TA, tubular adenomas; TVA, tubulovillous adenomas; VA, villous adenomas, SA, serrated adenomas; MS-PCR, Methylation-specific Polymerase Chain Reaction; q-MS-PCR, quantitative Methylation-specific Polymerase Chain Reaction; n-MS-PCR, nested Methylation-specific Polymerase Chain Reaction; MS-HRM, Methylation-sensitive High-resolution Melting; HM-450K., Human Methylation 450 k; SS, Screening Setting; CS, Clinical setting; DMR., Differentially Methylated Region; TSS, Transcriptional Start Site; SOMA, short oligonucleotide mass analysis.

**Supplementary Table S2.** DNA methylation difference between colorectal adenoma/ CRC cases and controls reported by P-value only.

| Gene/ LINE-1 locus | First author, Year [Ref. No.] | Country | Study Group | No. | Age (years) | DNA methylation assay | p-value* |
|---|---|---|---|---|---|---|---|
| **Hypomethylation** | | | | | | | |
| *IGF2* | Miroglio, 2010 [35] | France | CRC | 26 | 64 | Pyrosequencing | 0.045 |
| | | | Controls | 19 | 40 | | |
| *PDK4* | Leclerc, 2017 [42] | USA | Cohort 1: CRC | 40 | 59 | Pyrosequencing | <0.01 |
| | | | Controls | 40 | 58 | | |
| | | | Cohort 2: CRC | 18 | 60 | | <0.05 |
| | | | Controls | 29 | 60 | | |
| *SEPT9* | Ravegnini, 2015 [32] | Italy | CRC | 27 | 67.5 | MS-PCR | 0.0006 |
| | | | Controls | 26 | 59.6 | | |
| **Hypermethylation** | | | | | | | |
| *CNRIP1* | Zhang, 2017 [38] | China | Adenoma | 20 | 52.6 | q- MS-PCR, Pyrosequencing | 0.00 |
| | | | CRC | 50 | 69.4 | | 0.008 |
| | | | Controls | 20 | 49.2 | | |
| *ESR1* | Ally, 2009 [33] | UK | Adenoma | 30 | 65.5 | Pyrosequencing | > 0.05 |
| | | | CRC | 27 | 68.4 | | 0.17 |
| | | | Controls | 57 | 57.5 | | |
| *L1X5b* | Nüsgen, 2015 [36] | Lithuania | CRC | 21 | -- | Pyrosequencing | 0.04[a] |
| | | | Controls | 59 | | | |

[a] p-value reported for females only. * p-value represents difference of DNA methylation between cases and controls. Abbreviations: Ref., Reference; No., Number; CRC, colorectal cancer; MS-PCR, Methylation-specific Polymerase Chain Reaction; q- MS-PCR, quantitative Methylation-specific Polymerase Chain Reaction.

**Supplementary Table S3.** Methylation of genes with no significant association to colorectal adenoma/ CRC risk.

| Gene | First author, Year [Ref. No.] | Country | Study Group | No. | Mean Age (years) | DNAm assay | OR value (95%CI) | *p*-value |
|---|---|---|---|---|---|---|---|---|
| **Hypomethylation** | | | | | | | | |
| *APC* | Alexander, 2017 [43] | USA | Adenoma<br>Controls | 38<br>69 | -- | MS-PCR | 1.0 (0.4–2.5)[a, c] | 0.97 |
| *IGF2* | Ito, 2008 [34] | UK | Cohort 1: CRC<br>Controls | 225<br>435 | 66.1<br>66.1 | PS | 1.09 (0.65–1.82)[b, d] | 0.74 |
| *SFRP4* | Alexander, 2017 [43] | USA | Adenoma<br>Controls | 38<br>69 | -- | MS-PCR | 1.1 (0.3–3.8)[a] | 0.89 |
| *TIMP3* | Alexander, 2017 [43] | USA | Adenoma<br>Controls | 38<br>69 | -- | MS-PCR | 1.2 (0.4–3.0)[a] | 0.78 |
| *WIF1* | Alexander, 2017 [43] | USA | Adenoma<br>Controls | 38<br>69 | -- | MS-PCR | 1.3 (0.5–3.2)[a] | 0.56 |
| *TMEFF2* | Alexander, 2017 [43] | USA | Adenoma<br>Controls | 38<br>69 | -- | MS-PCR | 1.5 (0.6–3.4)[a] | 0.36 |
| *BRCA1* | Alexander, 2017 [43] | USA | Adenoma<br>Controls | 38<br>69 | -- | MS-PCR | 1.5 (0.6–3.9)[a] | 0.36 |
| *IGF2* | Ito, 2008 [34] | UK | Cohort 2: CRC<br>Controls | 192<br>96 | 65.9<br>43.2 | PS | 1.63 (0.06–42.5)[b, d] | 0.77 |
| *SFRP5* | Alexander, 2017 [43] | USA | Adenoma<br>Controls | 38<br>69 | -- | MS-PCR | 3.7 (0.8–17.1)[a] | 0.09 |
| **Hypermethylation** | | | | | | | | |
| *CRH* | Gao, 2018 [37] | China | CRC<br>Controls | 466<br>507 | 60.1<br>56.7 | MS-HRM | 0.54 (0.25–1.19)[e] | 0.13 |
| *PER2* | Alexander, 2017 [43] | USA | Adenoma<br>Controls | 38<br>69 | -- | MS-PCR | 0.6 (0.1–3.0)[f] | 0.56 |
| *ESR1* | Alexander, 2017 [43] | USA | Adenoma<br>Controls | 38<br>69 | -- | MS-PCR | 0.6 (0.3–1.5)[g] | 0.27 |
| *CDH1* | Liu, 2017 [39] | China | CRC<br>Controls | 428<br>428 | 59.4<br>59.4 | MS-HRM | 0.65 (0.36-1.19)[h] | 0.16 |

**Supplementary Table S3.** *Cont.*

| Gene | First author, Year [Ref. No.] | Country | Study Group | No. | Mean Age (years) | DNAm assay | OR value (95%CI) | *p*-value |
|---|---|---|---|---|---|---|---|---|
| *MLH1* | Alexander, 2017 [43] | USA | Adenoma<br>Controls | 38<br>69 | -- | MS-PCR | 0.7 (0.2–3.1)[i] | 0.67 |
| *DNMT3b (GCA, 31351216)* | Ho, 2015 [45] | Canada | Adenoma<br>Controls | 87<br>172 | -- | Sequenom EpiTYPER | 0.72 (0.31–1.65)[j, m] | 0.17 |
| *CYP27B1* | Alexander, 2017 [43] | USA | Adenoma<br>Controls | 38<br>69 | -- | MS-PCR | 0.8 (0.2–3.0)[i] | 0.72 |
| *IGF2* | Alexander, 2017 [43] | USA | Adenoma<br>Controls | 38<br>69 | -- | MS-PCR | 0.8 (0.3–2.0)[k] | 0.57 |
| *CDKN2A* | Alexander, 2017 [43] | USA | Adenoma<br>Controls | 38<br>69 | -- | MS-PCR | 0.8 (0.3–1.9)[g] | 0.54 |
| *NGFR* | Alexander, 2017 [43] | USA | Adenoma<br>Controls | 38<br>69 | -- | MS-PCR | 0.9 (0.3–2.2)[c] | 0.75 |
| *SEPT9* | Alexander, 2017 [43] | USA | Adenoma<br>Controls | 38<br>69 | -- | MS-PCR | 0.9 (0.4–2.1)[c] | 0.81 |
| *RARB2* | Luo, 2016 [40] | China | CRC<br>Controls | 421<br>506 | 59.5<br>56.6 | MS-HRM | 0.96 (0.70–1.30)[l] | 0.78 |
| *IGFII CpG2* | Kaaks, 2009 [28] | Sweden | CRC<br>Controls | 97<br>190 | 58<br>58 | SOMA | 1.00 (0.46–2.19)[m] | 0.58[n] |
| *IGFII CpG1* | Kaaks, 2009 [28] | Sweden | CRC<br>Controls | 97<br>190 | 58<br>58 | SOMA | 1.02 (0.49–2.16)[m] | 0.96[n] |
| *DNMT3b TSS* | Ho, 2015 [45] | Canada | Adenoma<br>Controls | 87<br>172 | -- | Sequenom EpiTYPER | 1.11 (0.49–2.51)[j, m] | 0.71 |
| *MTHFR GCA* | Ho, 2015 [45] | Canada | Adenoma<br>Controls | 181<br>88 | -- | Sequenom EpiTYPER | 1.11 (0.51–2.44)[j, m] | 0.96 |
| *DNMT3b (GCA, 31351122)* | Ho, 2015 [45] | Canada | Adenoma<br>Controls | 87<br>172 | -- | Sequenom EpiTYPER | 1.14 (0.49–2.42)[j, m] | 0.81 |
| *MTHFR TSS* | Ho, 2015 [45] | Canada | Adenoma<br>Controls | 181<br>88 | -- | Sequenom EpiTYPER | 1.17 (0.54–2.51)[j, m] | 0.89 |

**Supplementary Table S3.** *Cont.*

| Gene | First author, Year [Ref. No.] | Country | Study Group | No. | Mean Age (years) | DNAm assay | OR value (95%CI) | *p*- value |
|---|---|---|---|---|---|---|---|---|
| *DNMT3b Average* | Ho, 2015 [45] | Canada | Adenoma<br>Controls | 87<br>172 | -- | Sequenom EpiTYPER | 1.25 (0.54–2.86) [j, m] | 0.49 |
| *DNMT3b (GCA, 31351172-31351174)* | Ho, 2015 [45] | Canada | Adenoma<br>Controls | 87<br>172 | -- | Sequenom EpiTYPER | 1.32 (0.59–2.95) [j, m] | 0.57 |
| *FOXE-1* | Luo, 2016 [40] | China | CRC<br>Controls | 421<br>506 | 59.5<br>56.6 | MS-HRM | 1.35 (0.99–1.85) [l] | 0.06 |
| *MTHFR Average* | Ho, 2015 [45] | Canada | Adenoma<br>Controls | 181<br>88 | -- | Sequenom EpiTYPER | 1.45 (0.68–3.12) [j, m] | 0.47 |
| *TMEM132D* | Gao, 2018 [37] | China | CRC<br>Controls | 466<br>507 | 60.1<br>56.7 | MS-HRM | 1.52 (0.99–2.34) [e] | 0.06 |
| *DNMT3b (GCA, 31351072)* | Ho, 2015 [45] | Canada | Adenoma<br>Controls | 87<br>172 | -- | Sequenom EpiTYPER | 1.61 (0.67–3.88) [j, m] | 0.57 |
| *DNMT3b (GCA, 31350991 - 31350995)* | Ho, 2015 [45] | Canada | Adenoma<br>Controls | 87<br>172 | -- | Sequenom EpiTYPER | 1.65 (0.77–3.53) [j, m] | 0.11 |
| *MLH1* | Liu, 2017 [39] | China | CRC<br>Controls | 428<br>428 | 59.4<br>59.4 | MS-HRM | 1.72 (0.68-4.34) [h] | 0.25 |
| *APC* | Liu, 2017 [39] | China | CRC<br>Controls | 428<br>428 | 59.4<br>59.4 | MS-HRM | 1.82 (0.74-4.49) [h] | 0.19 |
| *DNMT3b (GCA, 31351226 - 31351230)* | Ho, 2015 [45] | Canada | Adenoma<br>Controls | 87<br>172 | -- | Sequenom EpiTYPER | 1.86 (0.83–4.15) [j, m] | 0.48 |
| *CDKN2A* | Liu, 2017 [39] | China | CRC<br>Controls | 428<br>428 | 59.4<br>59.4 | MS-HRM | 1.99 (0.56-7.04) [h] | 0.29 |
| *DNMT3b (GCA, 31351144)* | Ho, 2015 [45] | Canada | Adenoma<br>Controls | 87<br>172 | -- | Sequenom EpiTYPER | 1.99 (0.88–4.50) [j, m] | 0.37 |
| *APC* | Ashktorab, 2007 [44] | USA | AP/HPP Controls | 19<br>29 | 60.5<br>63.5 | MS-PCR | 3.6 (0.6–22.0) | 0.15 |

[a] Odds of adenoma development given no methylation detected in the candidate gene. [b] Odds of CRC development given loss of methylation detected in the candidate gene. OR: [c] Model adjusted for vitamin D and multivitamin use; [d] Model adjusted for BMI, number of packet smocked per year and family history of cancer; [e] Model adjusted for BMI, age, fruit, coarse grains, fruit can, pork intestines, fried food, garlic, and braised fish in brown sauce; [f] Model adjusted for vitamin D and multivitamin use, physical activity, and age group; [g] Model adjusted for vitamin D use, physical activity, and age; [h] Model adjusted for age, gender, BMI, occupational physical activity, smoking, and consumption of coarse grains, fish stewed with brown sauce, fried food, leftovers and pork; [i] Model adjusted for vitamin C and D use, physical activity, and age; [j] Model adjusted for sex and age; [k] Model adjusted for vitamin C and D use and physical activity; [l] Model adjusted for age, BMI, occupation and family history of cancer. [m] OR for Quartile 4 vs 1. [n] p-value reported for trend across quartiles of methylation level. Abbreviations: Ref., Reference; No., Number; DNAm, DNA methylation; CRC, colorectal cancer; AP, adenomatous polyp; HPP, hyperplastic polyp; MS-PCR, Methylation-specific Polymerase Chain Reaction; MS- HRM, Methylation-sensitive High-resolution Melting; PS, Pyrosequencing; SOMA, short oligonucleotide mass analysis.
